# Linear epitopes in *Onchocerca volvulus* vaccine candidate proteins and excretory‐secretory proteins

**DOI:** 10.1111/pim.12587

**Published:** 2018-10-08

**Authors:** Ole Lagatie, Ann Verheyen, Bieke Van Dorst, Linda Batsa Debrah, Alex Debrah, Lieven J. Stuyver

**Affiliations:** ^1^ Janssen Diagnostics Janssen R&D Beerse Belgium; ^2^ Department of Clinical Microbiology School of Medical Sciences Kwame Nkrumah University of Science and Technology Kumasi Ghana; ^3^ Faculty of Allied Health Sciences Kwame Nkrumah University of Science and Technology Kumasi Ghana

**Keywords:** excretome/secretome proteins, linear epitope, *Onchocerca volvulus*, onchocerciasis, river blindness, serology, vaccine

## Abstract

In our previous study, a proteome‐wide screen was conducted to identify linear epitopes in this parasite's proteome, resulting in the discovery of three immunodominant motifs. Here, we investigated whether such antigenic peptides were found in proteins that were already known as vaccine candidates and excretome/secretome proteins for *Onchocerca volvulus* This approach led to the identification of 71 immunoreactive stretches in 46 proteins. A deep‐dive into the immunoreactivity profiles of eight vaccine candidates that were chosen as most promising candidates for further development (*Ov*‐CPI‐2, *Ov*‐ALT‐1, *Ov*‐RAL‐2, *Ov*‐ASP‐1, *Ov*‐103, *Ov*‐RBP‐1, *Ov*‐CHI‐1, and *Ov*‐B20), resulted in the identification of a poly‐glutamine stretch in *Ov*‐RAL‐2 that has properties for use as a serodiagnostic marker for *O. volvulus* infection. A peptide ELISA was developed, and the performance of this assay was evaluated. Based on this assessment, it was found that this assay has a sensitivity of 75.0% [95% CI: 64.9%‐83.5%] and a specificity of 98.5% [95% CI: 94.6%‐99.8%]. Furthermore, 8.7% reactivity in Asian parasite‐infected individuals (8 out of 92) was observed. Besides this identification of a linear epitope marker, the information on the presence of linear epitopes in vaccine candidate proteins might be useful in the study of vaccines for river blindness.

## 1 BACKGROUND

Of the 20 infectious diseases listed on the World Health Organization (WHO) list of Neglected Tropical Diseases, eight of them are caused by a helminth infection.^1^, ^2^, ^3^ One of them, onchocerciasis (or river blindness) is caused by infection with the filarial nematode *Onchocerca volvulus*. The majority of infected people live in Africa, where at least 120 million are at risk.^4^, ^5^ Current treatment programs are based on mass drug administration (MDA) of the microfilaricidal agents ivermectin (Mectizan, Merck) as no approved macrofilaricide drugs or vaccines are available. However, contraindications in areas co‐endemic for loiasis, an inability to break transmission in some foci, and the possible emergence of drug resistance ask for a change in strategy including vaccination in order to control or eliminate onchocerciasis.^6^, ^7^

Two basic strategies were used to identify and clone *O. volvulus* target vaccine antigens: immunoscreening of various *O. volvulus* cDNA libraries, and rational antigen selection.^8^, ^9^, ^10^, ^11^, ^12^, ^13^, ^14^ Also, analysis of excretory‐secretory products (ESPs) in nodule fluid of cows infected with *Onchocerca ochengi* has led to the identification of 85 proteins with potential pharmacological properties or immunogenic potential.^15^ Based on a mouse‐*Onchocerca* model, the top ranking eight *Ov* protective antigens (*Ov*‐CPI‐2, *Ov*‐ALT‐1, *Ov*‐RAL‐2, *Ov*‐ASP‐1, *Ov*‐103, *Ov*‐RBP‐1, *Ov*‐CHI‐1, and *Ov*‐B20) were chosen for more extensive studies.^16^ Using two model systems, *O. volvulu* in mice and *Brugia malayi* in gerbils, this list of candidates has been reduced to the final selection of *Ov*‐103 and *Ov*‐RAL‐2 for further clinical development.^10^, ^16^, ^17^

In our previous work, the entire *O. volvulus* proteome was screened for the presence of linear epitopes.^18^ Based on this work, the serodiagnostic peptides OvMP‐1 and OvMP‐23 were proposed and evaluated as markers for onchocerciasis.^19^ In the study presented here, we use the previously obtained data from the high‐density peptide arrays to investigate whether the vaccine candidates and ESP's described above contain one or more peptide fragments that are recognized by antibodies in chronically infected individuals, whether immunodominant epitopes could be identified and whether some of them might have properties that make them attractive serodiagnostic candidates.

## 2 METHODS

### 2.1 Study samples

All samples used in this study were de‐identified before being provided, written informed consent was obtained from all individuals and usage of these samples for research purposes was approved by an ethical committee or Institutional Review Board (IRB).

A set of 12 samples from *O. volvulus* infected individuals, collected in Cameroon by Dr. Nutman, was obtained through the Filariasis Research Reagent Resource Center (FR3), Division of Microbiology and Infectious Diseases, NIAID, NIH. Information on *O. volvulus* infection (number of microfilaria/mg skin and number of palpable nodules) was provided by FR3, along with demographic information. All infected individuals had at least two palpable nodules and 25 mf/mg skin (microfilaridermia) as determined by skin snip. Sera were collected from clotted blood obtained by venipuncture. Detailed characteristics of the samples used was published before.^18^

Additionally, the second set of plasma samples from *O. volvulus* infected individuals were collected as part of a field study in Ghana. This study was undertaken in an Onchocerciasis‐endemic community located in Adansi South District along the Pra river basins in the Ashanti Region of Ghana. Physical examinations were performed to identify those subjects having palpable nodules. Skin snips (biopsies) were then taken in order to determine the microfilarial (mf) load in the skin.^20^ Most subjects were participating in mass drug administration programs. A total of 97 nodule positive subjects that donated plasma samples were included. Demographic information is provided in Table 1. For the non‐*Onchocerca* endemic control samples, demographic information is also provided in Table 1.

**Table 1:** Study populations used for determination of diagnostic performance

Characteristic	Group										
	*Onchocerca volvulus*	HC Southern Africa	HC Belgium	HIV	HCV	Asthma	Dengue	Malaria	*Wb*	*Bm*	STH
Origin	Ghana	Southern Africa	Belgium	USA	USA	USA	Vietnam	Vietnam	Sri Lanka (8) Tahiti (2)	Indonesia (Central Sulawesi)	Indonesia (Flores)
No. of patients	97	10	49	25	25	25	25	25	10	20	20
Age, median (Min‐Max)	47 (21‐85)	21 (17‐47)	40 (23‐59)	n.a.	n.a.	46(17‐91)	26 (4‐67)	22 (18‐40)	33 (13‐48)	19 (10‐45)	40 (25‐75)
Gender, n (%)											
Male	54 (56)	7 (70)	22 (45)	n.a.	n.a.	11 (44)	10 (40)	24 (96)	6 (60)	10 (50)	2 (10)
Female	43 (44)	3 (30)	27 (55)	n.a.	n.a.	14 (56)	15 (60)	1 (4)	3 (30)	10 (50)	18 (90)
Unknown	0 (0)	0 (0)	0 (0)	0 (0)	0 (0)	0 (0)	0 (0)	0 (0)	1 (10)	0 (0)	0 (0)
Source	KCCR	TS	Janssen	MC	MC	BR	DLS	DLS	FR3	UI	UI
Ov16 IgG4 positive, n	66	0	0	0	0	1	0	0	0	0	0

*Bm*:* Brugia malayi*; BR: Bioreclamation; DLS: Discovery Life Sciences; FR3: Filariasis Research Reagent Resource Center; KCCR: Kumasi Centre for Collaborative Research; MC: Mayo Clinic; n.a.: not available; STH: Soil‐transmitted helminths; TS: Tissue solutions; UI: Universitas Indonesia; *Wb*:* Wuchereria bancrofti*.

### 2.2 Peptide array analysis

Data of high‐density peptide arrays were obtained as described previously.^18^ For interpretation of the individual results, two cut‐offs were used, based on the previous analyses.^18^ A first cut‐off of 837 Relative Fluorescence Units (RFU) was used as the upper cut‐off for healthy control samples to be confirmed as negative. The second cut‐off of 4842 RFU was used as the lower cut‐off for samples from *O. volvulus* infected individuals to be confirmed as positive.

### 2.3 Total IgG peptide ELISA

Biotinylated synthetic peptide OVOC9988;25‐33 (QQQQQQQQR) was synthesized by standard procedures and purchased from PEPperPRINT GmbH (Heidelberg, Germany). For determination of peptide specific serum antibody levels, a peptide ELISA was developed as described previously.^18^

### 2.4 Bio‐IT analysis

Homologs of Ov‐RAL‐2 were identified using the BLAST tool in WormBase Parasite (Version WBPS10, WS263) and fasta files of the sequence of these homologs were obtained. Multiple Sequence Alignment was performed using the online Clustal W aligner tool in T‐COFFEE, Version_11.00.d625267 (http://tcoffee.crg.cat/apps/tcoffee/index.html).^21^ Identical or similar epitopes in human proteins were searched in the immune epitope database (http://www.iedb.org).^22^

### 2.5 Statistical analysis

For peptide ELISA, ROC analysis was performed. Several sample sets from non‐helminth infected western individuals were used as control group. These include healthy controls from both Belgium and South Africa, HIV‐infected or HCV‐infected individuals from USA, and asthma patients from the USA. Samples from *O. volvulus* infected individuals collected in Ghana were used as positive group. ROC analysis was performed using all non‐helminth controls vs *Ov* positive samples. A cut‐off was determined as the average response in all samples from the control group + 2 times the standard deviation of these responses (ie, 0.082). Based on this cut‐off, sensitivity and specificity was determined, as well as cross‐reactivity with other helminths. All analyses were performed using GraphPad Prism 7 (La Jolla, CA, USA).

## 3 RESULTS

### 3.1 Linear epitopes in eight selected vaccine candidate proteins

**Figure 1:**
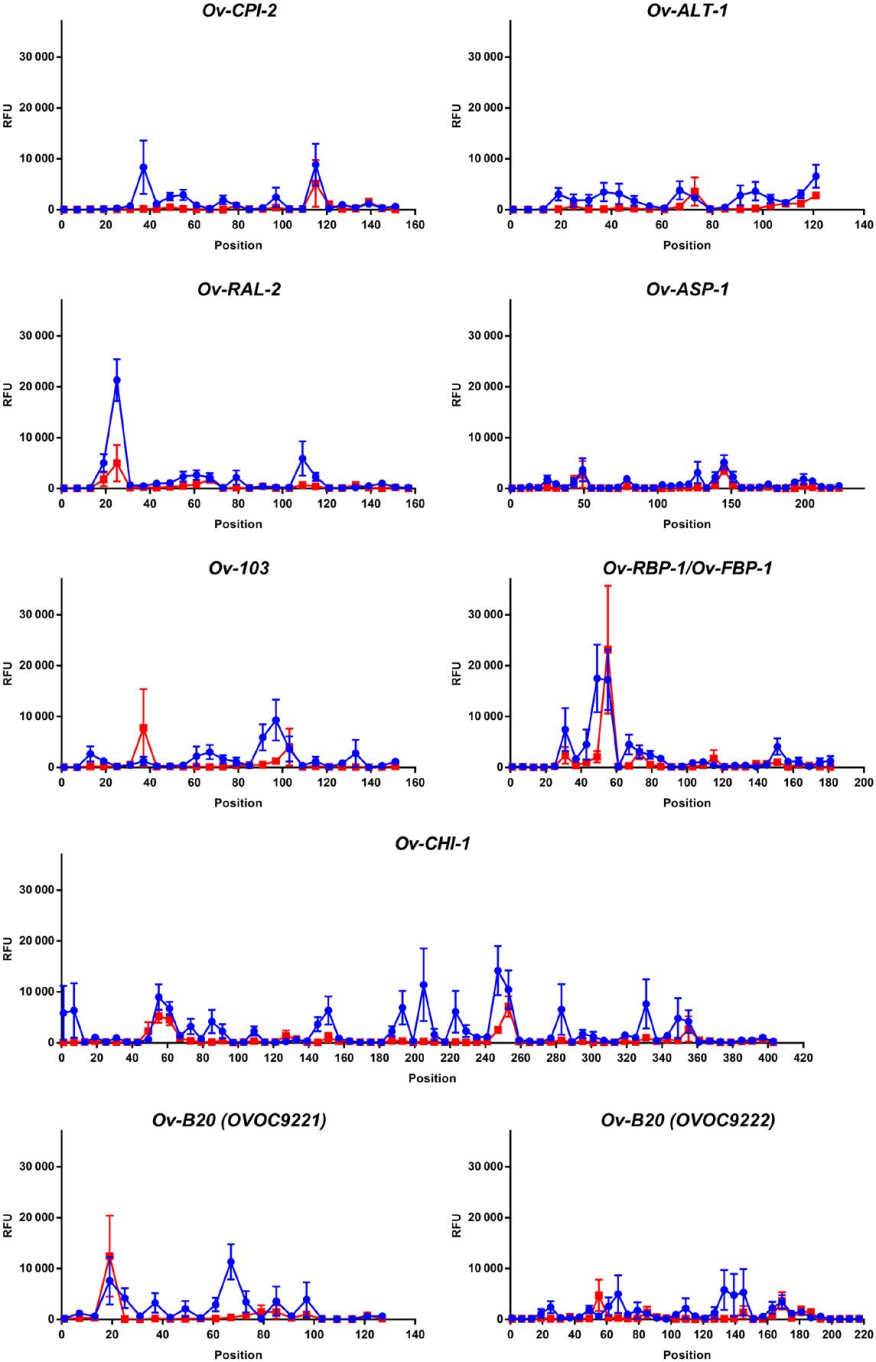
Response against peptides derived from the eight selected vaccine candidate proteins *Ov*‐CPI‐2, *Ov*‐ALT‐1, *Ov*‐RAL‐2, *Ov*‐ASP‐1, *Ov*‐103, *Ov*‐RBP‐1, *Ov*‐CHI‐1 and *Ov*‐B20. For every peptide derived from these *Onchocerca volvulus* proteins signals in the peptide arrays are plotted as the average of 12 infected individuals (blue circles) and of six healthy controls (red squares). Error bars represent SEM. Numbers in the *x*‐axis indicate the start position of the peptide with reference to the full‐length protein

In 2015, an international consortium launched The Onchocerciasis Vaccine for Africa (TOVA) with the goal of evaluating and pursuing vaccine development for onchocerciasis. After a rational design for the antigen discovery and selection process, eight recombinant proteins were selected for preclinical development: *Ov*‐CPI‐2, *Ov*‐ALT‐1, *Ov*‐RAL‐2, *Ov*‐ASP‐1, *Ov*‐103, *Ov*‐RBP‐1, *Ov*‐CHI‐1, and *Ov*‐B20.^16^ The corresponding native proteins are encoded by the genes OVOC7453, OVOC12769, OVOC9988, OVOC9575, OVOC4230, OVOC8754, OVOC12569, and OVOC9221/9222, respectively. All peptides used in the high‐density peptide array from these proteins were listed, and signals of the onchocerciasis patients and healthy controls were plotted (Figure 1 and Data S2). Detailed investigation of the immune response against peptides from these eight proteins shows that immunodominant linear epitopes are present in these proteins. A peptide was considered to bear immunodominant epitopes if at least 8 out of 12 cases had a signal above 4842 RFU. In *Ov*‐RAL‐2 there is a poly‐glutamine stretch between residues 25 and 33 (QQQQQQQQR) that is recognized by 9 of the 12 infected individuals. Also in *Ov*‐B20 (OVOC9221) one peptide between residues 67 and 80 (WATTENSSSIDSNN) was found to be recognized by 9 of the 12 infected individuals, but an average signal of the onchocerciasis cases was markedly lower than for the peptide from *Ov*‐RAL‐2. It must, however, be said that the poly‐glutamine stretch in *Ov*‐RAL‐2 is also recognized by one of the six healthy controls on the peptide arrays. In the case of *Ov*‐103 there is a region between residue 91 and 113 (FRETMSNPKMDFTNKENKWE) that is recognized by 8 of the 12 infected individuals and by none of the healthy controls. Antibodies in the individual samples appear to recognize either the first or the last part of this region with 3 out of 12 positive for peptide FRETMSNPKMDFTN and 6 out of 12 positive for peptide NPKMDFTNKENKWE. An increase in the response on the array is however, also observed in the healthy controls for either of both peptides, albeit markedly lower than in the infected individuals.

### 3.2 Peptide ELISA based on poly‐glutamine stretch of Ov‐RAL‐2

**Figure 2:**
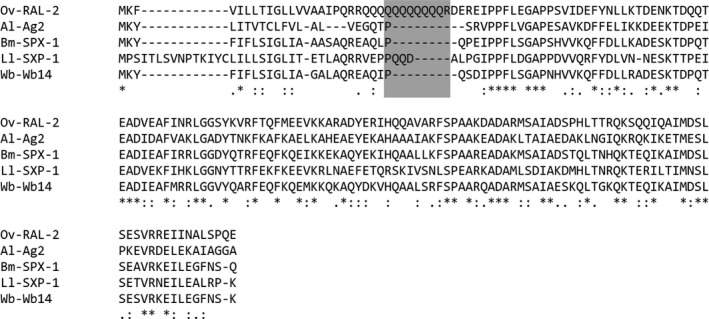
Alignment of Ov‐RAL‐2 (OVOC9988) and its closely related homologs in other helminth species (*Brugia malayi, Wuchereria bancrofti, L. loa* and *Ascaris lumbricoides*). The location of the immunodominant peptide has been indicated by the grey box

Based on the strong and dominant immune response that was observed against the poly‐glutamine stretch in *Ov*‐RAL‐2, and the fact that the same stretch was identified as immunoreactive peptide in a previous study, we decided to further explore this peptide.^23^ When investigating the closely related homologs of the *O. volvulus* vaccine candidates in other helminth species, we found that the poly‐glutamine stretch in the N‐terminal region of *Ov*‐RAL‐two is absent in all homologs from the other species, such as SPX‐1 in *B. malayi*, Wb14 in *Wuchereria bancrofti*, SXP‐1 protein in *Loa loa,* or Ag2 in *Ascaris lumbricoides* (Figure 2). The lack of this stretch in its closely related homologs makes this peptide very attractive for further diagnostic development as chances of cross‐reactivity with other helminth parasites are consequently expected to be low. Blast analysis of the peptide sequence (QQQQQQQQR) against all sequences available in the NCBI database, however, returned hundreds of proteins that also contain this peptide stretch, also in human proteins (results not shown). One of them was the protein Bm6219 (also known as Bma‐ttn‐1), a large Immunoglobulin I‐set domain containing protein that is closely related to OVOC7180, an *O. volvulus* ESP and vaccine candidate. In OVOC7180, however, this peptide stretch is not present. Exploration of the immune epitope database for known human (autoimmune related) epitopes did not return any identical hit for this peptide sequence, but one epitope in the human protein Trinucleotide repeat‐containing gene 6A was found to have a similar poly‐glutamine stretch.

**Figure 3:**
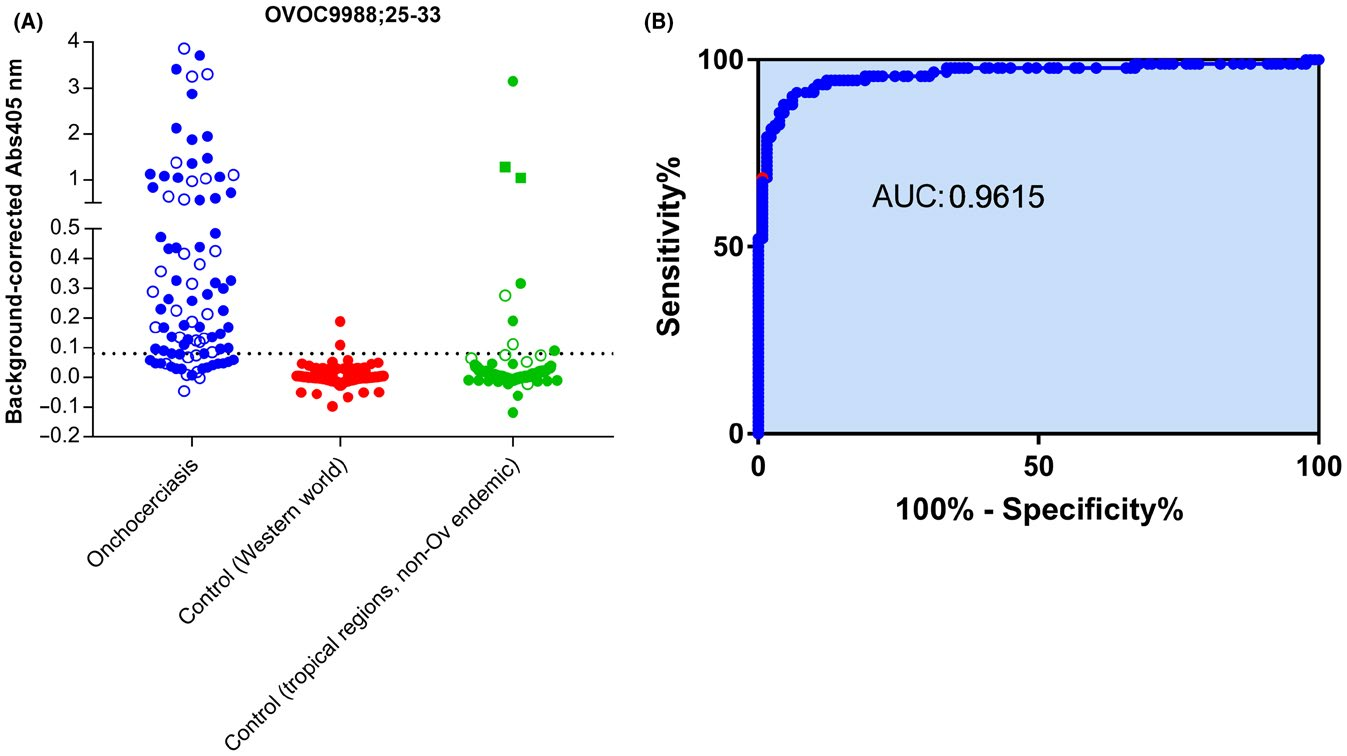
Peptide ELISA based assessment of immunoreactivity against OVOC9988;25‐33. A, Immunoreactivity against OVOC9988;25‐33 was determined in *Onchocerca volvulus* infected individuals (● Ov16 RDT positive, ○ Ov16 RDT negative), controls from Western world and controls from non‐*Ov* endemic tropical regions that are known to have other parasitic infections (● Vietnam, ○ Indonesia, ■ Tahiti, ▲ Sri Lanka). Dotted line indicates the cut‐off for positivity. B, ROC analysis of the peptide ELISA with OVOC9988;25‐33 as antigen using onchocerciasis as cases and all Western controls as controls

In order to confirm the observation from the high‐density peptide array and to further study the specificity of this peptide, we developed a peptide ELISA based on this 9‐mer peptide and determined immune reactivity against this peptide in a set of 92 nodule positive individuals and in several sets of non‐*Onchocerca* infected individuals (Figure 3). These peptide ELISA data confirm the response in onchocerciasis patients, but also demonstrate that the one positive healthy control sample on the peptide arrays was most likely a technical artefact as none of the healthy controls (both seen and unseen) had a response above the cut‐off. Based on this data set, this peptide has an area under curve of 0.9615 in ROC analysis, a sensitivity of 75.0% [95% CI: 64.9%‐83.5%] and a specificity of 98.5% [95% CI: 94.6%‐99.8%]. Of interest, 25 of the 31 (80.6%) Ov16 RDT negative samples were found to be positive for the poly‐glutamine peptide (Figure 3). The data also demonstrate that there is reactivity in 8.7% of the individuals from Asia (8 out of 92), who had known infections with other parasites such as malaria, lymphatic filariasis and soil‐transmitted helminths (STH). Most pronounced responses in this group were observed in the two individuals from Tahiti (infected with *W. bancrofti*) and one of the 50 individuals from Vietnam (who had a Malaria infection).

### 3.3 Detection of linear epitopes in extended list of vaccine candidates and ESP's

**Figure 4:**
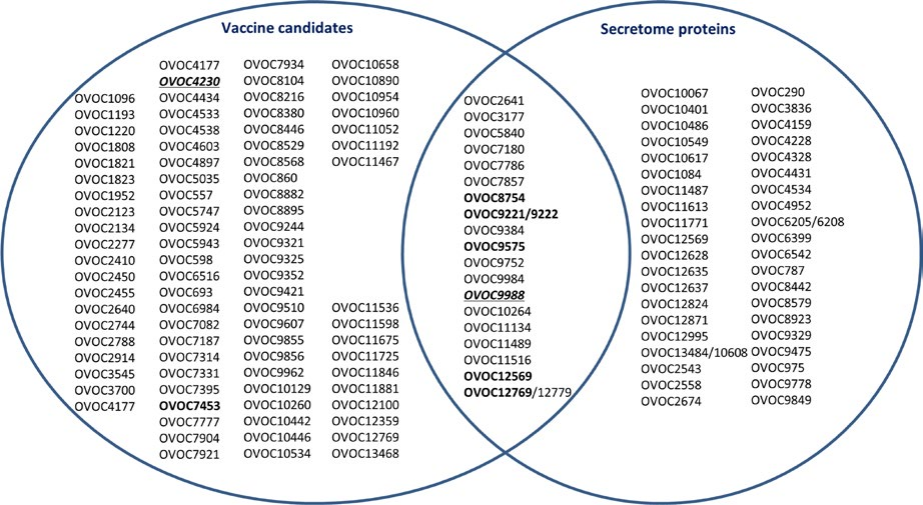
Venn diagram showing the overlap between proteins found in the list of vaccine candidates (left) and excretory‐secretory products (ESP's) (right) of *Onchocerca volvulus*. The eight proteins that were selected for further preclinical evaluation are indicated in bold; the two proteins that have been selected for clinical evaluation as vaccine are indicated in italics and underlined

Since the analysis on the eight selected vaccine candidates demonstrated that immunodominant linear epitopes are present in these proteins, we explored the entire list of vaccine candidates and ESP's for the presence of such immunodominant linear epitopes. Based on the work published by others, and using the Blast tool in WormBase ParaSite, two lists with OVOC ID's were generated (Data S1): (a) 105 proteins suggested as vaccine candidates; and (b) 58 proteins for which fragments were found in the excretory fluid of *O. ochengi*.^8^, ^9^, ^10^, ^11^, ^12^, ^13^, ^14^, ^15^, ^16^, ^17^ In case Blast did not result in unambiguous identification of the OVOC ID, both hits were listed. A Venn diagram was prepared of these lists (containing a total of 146 proteins) to identify proteins that overlap between the two lists (Figure 4). Subsequently, all peptides used in the high‐density peptide array from these proteins were listed, including the response in the arrays for the individual samples on these peptides (Data S2).

Based on the immunoreactivity table of all peptides from vaccine candidate proteins and ESP's, we searched for peptides that had a detectable signal in no more than one of the six healthy controls (RFU <837) and for which at least 6 of the 12 infected individuals were clearly positive (RFU >4842). This resulted in a set of 71 peptides that had a signal in at least half (ie, 6 out of 12 infected individuals) (Data S2). For 32 vaccine candidates and 24 ESP's, peptides were found that were recognized by at least half of the infected individuals, 10 of those proteins being present in both groups. The most pronounced responses were observed for OVOC9384. For this protein‐10 peptides were found that were recognized by at least six individuals, one1 of them being recognized by 11 individuals. Interestingly, three of those peptides also consisted of poly‐glutamine stretches, but with one or two other amino acids in between. Whether these stretches in OVOC9384 are responsible for inducing the immune response or the poly‐glutamine stretch in OVOC9988 cannot be deduced from these data. OVOC9384 is known as Gln‐rich protein and has been proposed by others as serodiagnostic marker for onchocerciasis.^24^ Based on our observation here, it appears that linear epitopes in OVOC9384 play an important role in the immune recognition of this protein.

## 4 DISCUSSION

From the longlist of vaccine candidates for *O. volvulus*, ultimately 8 proteins have been selected for preclinical evaluation and two of them, *Ov*‐103 and *Ov*‐RAL‐2, were picked for further clinical development as vaccine candidate.^12^, ^16^ In the study presented here, we investigated in detail the immunoreactivity of peptides derived from these eight proteins. Several peptide stretches in all of these proteins were found that were recognized by a small number of infected individuals (less than half of them). In *Ov*‐103 and *Ov*‐RAL‐2, the two candidates in clinical development, it was observed that a stretch is present that is recognized by eight or nine of the 12 infected individuals, respectively. Although we only confirmed the results from high‐density peptide array in peptide ELISA for the stretch in *Ov*‐RAL‐2, our data indicate that these proteins are obviously already exposed to the immune system upon natural infection and an immune response is being raised against a particular linear epitope in these proteins but clearly this is not able to stop further development of the parasite as the onchocerciasis patients used to perform the high‐density peptide arrays had high microfilarial load.^18^

Given the large portion of infected individuals that had a strong response to the 9‐mer peptide from *Ov*‐RAL‐2 with sequence QQQQQQQQR and the fact that this sequence was identified before as immunoreactive peptide, we investigated whether this peptide could be an interesting serological marker for infection with *O. volvulus*.^23^ Of interest, the poly‐glutamine stretch in the N‐terminal region of *Ov*‐RAL‐2, is absent in all its closely related homologs in other helminth species making this peptide very attractive for further diagnostic development. Blast analysis however also showed that this small peptide stretch is found in many proteins, one of them Bm6219, a close homolog of OVOC7180. Since both the *B. malayi* and the *O. volvulus* protein are known to be ESPs, it cannot be excluded that an immune response against this stretch can also be induced by exposure to the *B. malayi* protein in filariasis patients.^15^, ^25^ The fact that this peptide stretch is present in human proteins as well is of interest as this would mean that in uninfected individuals there should be immune tolerance towards this sequence which is abrogated upon infection with *Onchocerca*. Based on the information in the immune epitope database this sequence is similar to an epitope in Trinucleotide repeat‐containing gene 6A (also called TNRC6A or GW182), a protein that is involved in autoimmunity. It is however not clear whether this specific epitope also is involved in autoimmunity as research on this protein shows that other epitopes seem to play a role in its autoimmunity.^26^

A peptide ELISA was developed with this peptide and a larger panel of onchocerciasis patients and non‐*Onchocerca* infected individuals was evaluated. These data confirmed that this peptide is indeed recognized by a large portion of *O. volvulus* infected individuals (75.0%) while the majority of non‐*Onchocerca* infected individuals did not. 98.5% of non‐helminth infected individuals from the Western world and 91.3% of individuals from Asia and infected with other parasites had no detectable antibodies against this peptide. A strong response was however detected against this peptide in the two samples from *W. bancrofti* infected individuals that were collected in Tahiti and in one sample from a Malaria patient in Vietnam. Although there are no reports on *O. volvulus* occurrence in French Polynesia, to which Tahiti belongs, neither in Vietnam, it has been described that *Onchocerca. lupi*, the closely related species that infects dogs and that is transmitted by the same vector, occurs in that region and can even lead to zoonotic infection in humans.^27^, ^28^, ^29^, ^30^ It can therefore not be excluded that the response we detected in those individuals was in fact induced by exposure to *O. lupi*. More studies in non‐endemic but also *O. volvulus* hypo‐ and meso‐endemic areas will be needed to better understand the specificity profile of this peptide serology marker. The fact that 80.6% of the onchocerciasis patients with negative Ov16 RDT were found to be positive for this poly‐glutamine peptide makes it an attractive complement for this serological test. Eventually, this peptide could be combined with other *O. volvulus* derived peptides to make up a classifier, an approach that was shown before to be of value to monitor parasite persistence in Chagas’ Disease.^31^

Since the analysis on the eight selected vaccine candidates resulted in the identification of immunodominant linear epitopes, we also explored the entire list of vaccine candidates and ESP's for the presence of such immunodominant linear epitopes. A first observation we made during this analysis is that six of the vaccine candidate proteins that have been selected in the shortlist of 8 are also found in nodule fluid, indicating that they are part of the ESPs of the parasite.^10^, ^16^ Second observation is that 32 out of the 105 investigated vaccine candidate proteins have linear epitopes that are specifically recognized by at least half of the infected individuals as measured using high‐density peptide arrays. Also in the ESPs we found that of the 58 proteins 24 contained immunodominant linear epitopes (ie, peptide stretches that are recognized by at least half of the infected individuals). None of the linear epitopes found were recognized by all infected individuals. It is not clear why not all infected individuals raised an immune response against the linear epitopes found in both the vaccine candidate and ESPs. A difference in HLA type of the infected individuals might be responsible for this,^32^ but for ESPs it might also be possible that some nodules disseminate more of these proteins in the periphery (‘leaky nodules’), making them more accessible for the immune system.

In conclusion, this work demonstrates that several vaccine candidate proteins and excretome/secretome proteins display immunodominant linear epitopes. We also demonstrate that a 9‐mer peptide containing a poly‐glutamine stretch from the vaccine candidate *Ov*‐RAL‐2 might be an interesting serological marker warranting further exploration.

## CONFLICT OF INTEREST

OL, AV, BVD and LJS are current employees of Janssen Pharmaceutica NV, a Johnson and Johnson Company and may own stock or stock options in that company.
